# The association of serum choline concentrations with the risk of cancers: a community-based nested case–control study

**DOI:** 10.1038/s41598-023-49610-3

**Published:** 2023-12-13

**Authors:** Wenqiang Li, Chong Li, Tong Liu, Yun Song, Ping Chen, Lishun Liu, Binyan Wang, Jun Qu

**Affiliations:** 1 Department of General Surgery, Aerospace Center Hospital, Beijing, 100038 China; 2 Department of Oncology, Dazu Hospital of Chongqing Medical University, Chongqing, 402360 China; 3 Department of Gastrointestinal Surgery/Clinical Nutrition, Capital Medical University Affiliated Beijing Shijitan Hospital, Beijing, 100038 China; 4 Shenzhen Evergreen Medical Institute, Shenzhen, 518000 China

**Keywords:** Cancer, Oncology, Risk factors

## Abstract

Few studies have been designed to investigate the effect of serum choline on the risk of incident cancer. This study aims to explore the association between serum choline and the risk of new-onset cancer. We conducted a case–control study, including 199 patients with incident cancer and 199 matched controls during a median of 3.9 years of follow-up, nested within the China Stroke Primary Prevention Trial. Cubic spline regression (RCS) and conditional logistic regression analysis was used to assess the association of serum choline and incident cancer risk. We observed a positive dose–response association between serum choline levels and the risk of overall (*p* for overall = 0.046) and digestive system cancer (*p* for overall = 0.039). Compared with patients with the lowest choline levels (Q1 group), patients in the highest levels of choline (Q4) had a 3.69-fold and 6.01-fold increased risk of overall (OR = 3.69, 95% CI 1.17–11.63) and digestive system cancer (OR = 6.01, 95% CI 1.14–31.67). Elevated choline levels (per SD, 11.49 μg/mL) were associated with a higher risk of overall cancer among participants who were older, male, and smokers in the subgroup analyses. We found a positive association between elevated levels of serum choline with increased risk of incident cancer. Our findings have critical clinical implications for cancer prevention and diagnosis.

*Trial registration* CSPPT, NCT00794885. Registered: November 20, 2008. https://www.clinicaltrials.gov/ct2/show/study/NCT00794885https://www.clinicaltrials.gov/ct2/show/study/NCT00794885.

## Introduction

Cancer is the second leading cause of death globally and was responsible for an estimated 9.6 million deaths in 2018^1^. With the burden of cancer rising worldwide, robust scientific evidence is crucial for understanding its causes and prevention, and for early diagnosis. Despite the lack of a known fundamental cause, several predisposing conditions are recognized, including family history, infectious agents, tobacco use, diet, lack of exercise, chronic diseases, as well as environmental factors^2–7^.

As an essential nutrient, choline is vital for its many important biological functions. Firstly, choline, along with folate and other B vitamins, functions as a methyl donor and together they comprise what is known as one-carbon metabolism^8^. Secondly, choline serves as a precursor of acetylcholine, which is the principal neurotransmitter in all autonomic ganglia^8^. Thirdly, choline is a major component of the cell membrane structure (as the membrane phospholipids: phosphatidylcholine and sphingomyelin)^9^. Fourthly, choline also plays an important role in supporting fetal growth and development^10^. Even though choline can be produced endogenously in the liver, for most people, it needs to be obtained from the diet^11^. Studies have shown that a choline-deficient diet can lead to an increased risk of liver damage, fatty liver disease, and cognitive decline^12,13^.

As part of the one-carbon metabolism, choline plays an important role in constructing an integrated biochemical network to transfer one-carbon (methyl) groups^14^. Eventually, DNA methylation occurs when a methyl group is donated. Alterations in gene function occur as a result of DNA methylation, without creating deviations to the DNA sequence. DNA methylation is involved with gene expression and is critical for the development of cancerous cells^15^. Emerging evidence has revealed an association between the effect of dietary choline and serum choline on the risk of incident cancer. In case–control studies, dietary choline intake has been shown to be associated with a decreased risk of breast cancer^16^, colorectal cancer^17^, and liver cancer^18^. However, a reversed association between serum choline and colorectal cancer risk has also been observed in nested case–control studies^19,20^.

Given the lack of studies on the relationship between serum choline and subsequent cancer risk, this study aims to explore the association by using data from the China Stroke Primary Prevention Trial (CSPPT).

## Methods

### Study population

Data for this nested case–control study were drawn from the CSPPT, whose methods and main results have been described previously^21^. The CSPPT aimed to explore whether a combined treatment of enalapril maleate and folic acid was more effective in preventing stroke among primary hypertensive patients than enalapril maleate alone. From 19 May 2008 to 24 August 2013, a multi-center, randomized, double-blind clinical trial was conducted among 20,702 hypertensive patients (aged 45–75 years) in China. Hypertension was defined by a seated systolic blood pressure (SBP) of ≥ 140 mmHg, a diastolic blood pressure (DBP) of ≥ 90 mmHg, or the use of antihypertensive medication at the screening visit. Participants with a history of myocardial infarction, stroke, congenital heart disease, or heart failure were excluded at baseline. They were randomly assigned to one of two treatment groups in a 1:1 ratio: the enalapril-folic acid group (taking one 10 mg enalapril and 0.8 mg folic acid tablet once daily orally) or the enalapril group (taking one 10 mg enalapril tablet once daily orally). Every 3 months, a follow-up was conducted for each participant, during which drug adherence, concomitant medication usage, and other events of interest were collected and recorded by trained medical staff. Primary outcomes included stroke, cardiovascular events, all-cause death, myocardial infarction, and malignant tumors. Both the CSPPT and the present study received approval from the Ethics Committee of the Institute of Biomedicine, Anhui Medical University, Hefei, China (FWA assurance number: FWA00001263). The study is also registered at ClinicalTrials.gov under the identifier NCT00794885. All participants or their representative relatives provided written, informed consent.

### Outcome assessment

Cancer cases were diagnosed based on either specific clinical features or positive histopathologic results from the hospitals where patients had received treatment for malignant tumors. When pathological results were unavailable, potential cases were further evaluated by two oncologists, and a diagnosis was confirmed only when both clinicians agreed. Members of an independent Endpoint Adjudication Committee, who were unaware of study-group assignments, also reviewed and adjudicated all cancer events.

### Nested case–control study

**Figure 1:**
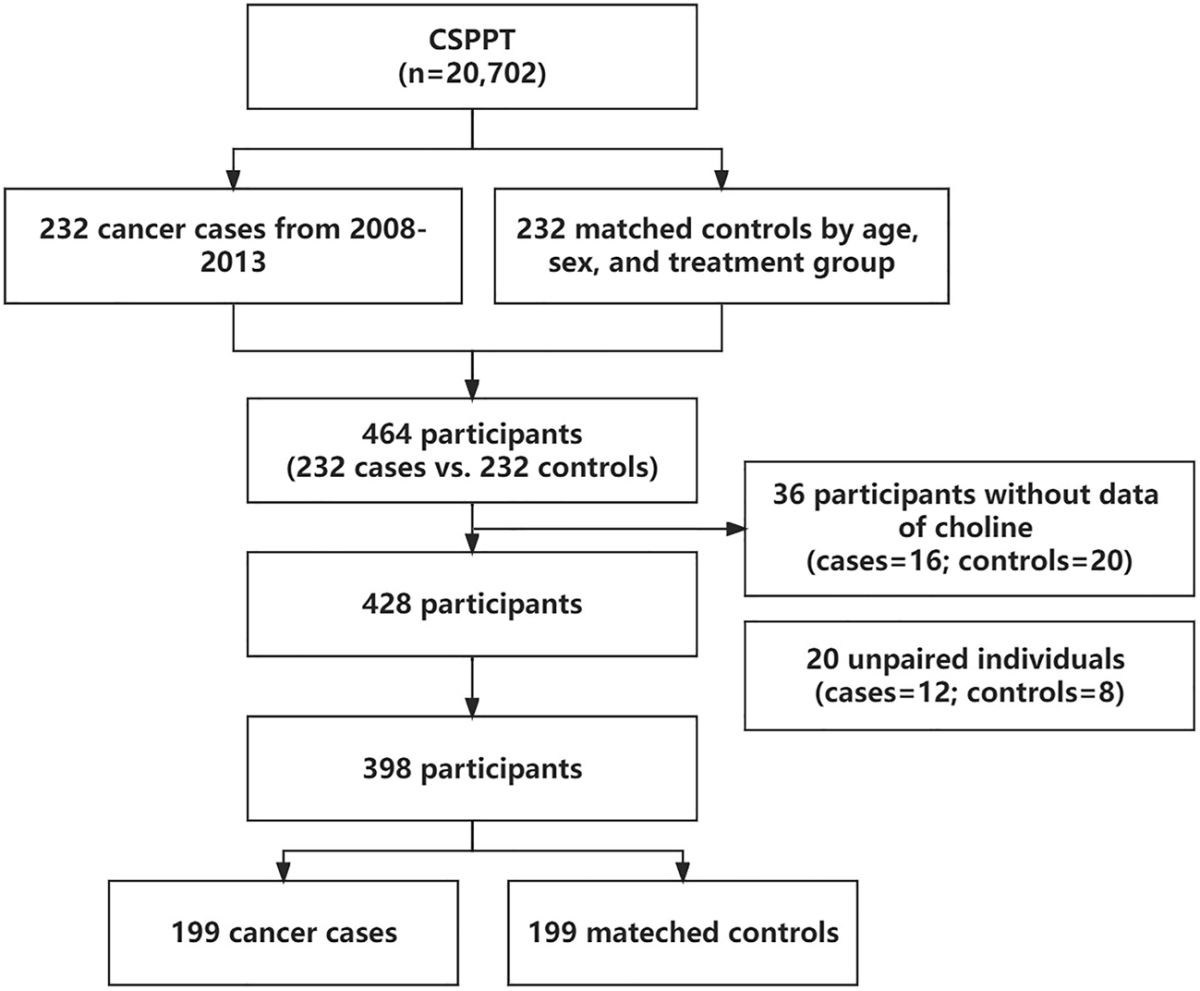
Flow chart of study participants in the nested case–control study within the China Stroke Primary Prevention Trial, conducted between May 2008 and August 2013.

During the CSPPT follow-up, 232 cancer cases occurred. For these cases, a nested case–control study was conducted, involving 232 new-onset cancer cases and 232 matched controls from the same district. Controls were chosen from participants who had not developed cancer during the follow-up. They were matched to the cases in a 1:1 ratio based on age (± 1 year), sex, and treatment group. After excluding patients with missing serum choline measurements (n = 36, including 16 cases and 20 controls) and unpaired individuals (n = 20, including 12 cases and 8 controls), the final analysis included a total of 398 participants, comprising 199 cancer cases and 199 matched controls (Fig. 1).

### Potential confounders

At the baseline screening in May 2008, we gathered information regarding participants' socioeconomic background and lifestyle habits, such as smoking and drinking status, using a standard questionnaire. Skilled medical staff carried out all participants' body measurements. After an overnight fast, we collected morning serum samples from all patients. We detected C677t gene (rs1801133) polymorphisms of 5,10-methylenetetrahydrofolate reductase (MTHFR), a genetic determinant of plasma homocysteine (HCY) levels, using an ABI Prism 7900HT sequence detection system (Life Technologies) and the TaqMan assay. We measured serum HCY and creatinine using the cycling enzymatic method, analyzed fasting blood glucose (FBG) with the hexokinase/glucose-6-phosphate dehydrogenase method, and measured both triglycerides (TG) and total cholesterol (TC) using the enzymatic colorimetric method. We determined serum high-density lipoprotein cholesterol (HDL-C) using the direct test method, serum uric acid (SUA) with the oxidase method, and serum folate using a chemiluminescent immunoassay (New Industrial). We analyzed biochemical indexes with automatic clinical analyzers (Beckman Coulter) at the central laboratory of the National Clinical Research Center for Kidney Disease, Nanfang Hospital, Guangzhou, China. We measured serum choline using liquid chromatography with tandem quadrupole mass spectrometry (LC–MS/MS) at a commercial lab, Beijing DIAN Medical Laboratory, China. The intra-assay and inter-assay coefficient of variation for each measurement was less than 10%.

### Statistical analysis

Continuous variables with normal distribution were presented as means ± SD and compared using generalized paired t-tests. Variables with a skewed distribution included SUA, creatinine, folate, HCY and choline, and these were presented as median ± IQR, and analyzed using nonparametric Kruskal–Wallis tests. Categorical variables were presented as n (%) and compared using χ2 tests. The dose–response association (non-linear and overall) is calculated by restricted cubic spline regression (RCS) with the reference set at the median value of serum choline. In our analysis, *p*-overall denotes the significance of the entire model, assessing the impact of all independent variables on the dependent variable. Meanwhile, *p*-nonlinear signifies the importance of adding nonlinear terms to the model. This analysis considers factors such as BMI, treatment category, tobacco use, alcohol consumption, MTHFR C677T, blood pressure levels, folate levels, triglyceride levels, cholesterol levels, uric acid levels, glucose levels, and homocysteine levels. Patients are categorized into four subgroups according to quartiles of serum choline at baseline: quartile 1 (< 5.35 μg/mL), quartile 2 (5.35–14.70 μg/mL), quartile 3 (14.71–25.18 μg/mL), and quartile 4 (≥ 25.19 μg/mL). Odds ratios (ORs) and 95% confidence intervals (CIs) are calculated by conditional logistic regression models to assess the association between serum choline (per SD increase and quartiles) and the risk of incident cancer. Multivariate analysis includes BMI, treatment group, smoking status, alcohol drinking, MTHFR C677T, systolic blood pressure, folate, triglycerides, cholesterol, uric acid, glucose, and homocysteine. Similar analysis is also conducted for digestive system cancer and non-digestive system cancer.

In addition, potential modifiers of the association between choline (per SD) and cancer are assessed by subgroup analyses on variables such as sex (men vs. women), age (median, < 65 years vs. ≥ 65 years), BMI (< 24.0 vs. ≥ 24.0 kg/m2), treatment group (enalapril vs. enalapril-folic acid), MTHFR c677T (CC + CT vs. TT), the median of folate (median, < 9.00 ng/mL vs. ≥ 9.00 ng/mL), smoking status (no vs. past/current), and drinking status (no vs. past/current). Conditional logistic regression models are utilized for subgroup analyses, accounting for the relevant covariates as adjusted in the logistic model for the total population. However, the variables used for stratification in each subgroup analysis are excluded from the adjusted covariates. Potential interactions between choline and these variables are examined by multiplicative models.

Considering the typically slow development of malignant tumors, if the follow-up time between baseline screening and diagnosis is short, there's a higher probability that undetected, undiagnosed precancerous lesions or occult cancer existed at the baseline sampling. To account for this, we further exclude those cancer cases that occurred within the first year of follow-up in a sensitivity analysis to eliminate the possibility of reverse causation.

All statistical computations are performed using a commercially available software program (SAS software, version 9.4). A two-tailed *p* < 0.05 is considered statistically significant in all analyses.

### Ethical approval

The CSPPT and the present study were approved by the Ethics Committee of the Institute of Biomedicine, Anhui Medical University, Hefei, China (FWA assurance number: FWA00001263) and is registered with ClinicalTrials.gov, NCT00794885. Written, informed consent was obtained from all participants or their representative relatives.

## Results

### Characteristics of the study population

The participants had a mean age of 62.12 ± 6.74 years, comprising 212 males (53.27%) and 186 females (46.73%). This study included 199 cancer cases. For all participants, the median serum choline level was 14.71 with an IQR of 5.35 to 25.19. The baseline characteristics for cases and controls are presented in Table 1. Cancer patients were more likely to smoke and had higher concentrations of serum choline compared to matched controls. There were no significant differences in age, BMI levels, SBP, DBP, TC, TG, SUA, HDL-C, FBG, creatinine, folate, HCY, or choline between cases and matched controls. Additionally, there were no significant differences in sex, treatment group, MTHFR c677t, or drinking status between the two groups (*p* value > 0.05).

**Table 1:** Baseline characteristics of the cases and matched controls.

Variables	Controls (n = 199)	Cases (n = 199)	*p*-value
Age, y	62.12 ± 6.74	62.11 ± 6.75	0.986
Male, n (%)	106 (53.27)	106(53.21)	1.000
BMI, kg/m^2^	24.17 ± 3.75	23.95 ± 3.49	0.534
Baseline SBP, mmHg	165.09 ± 16.74	163.81 ± 18.68	0.478
Baseline DBP, mmHg	93.51 ± 12.11	92.21 ± 11.77	0.278
TG, mmol/L	1.31(1.05,1.86)	1.40(1.04,1.93)	0.887
TC, mmol/L	5.31 ± 1.11	5.30 ± 1.15	0.920
SUA, mg/dL	295(252,348)	304(249,355)	0.635
HDL-C, mmol/L	1.30 ± 0.33	1.33 ± 0.35	0.427
FBG, mmol/L	5.73 ± 1.93	5.50 ± 1.55	0.215
Creatinine, mmol/L	66.80 (56.10,81.40)	68.00(57.20,77.10)	0.864
Folate, ng/mL	8.56 (6.12,11.66)	8.86 (6.03, 11.36)	0.994
HCY, μmol/L	12.87 (10.96,16.54)	12.77 (10.73,15.52)	0.497
Choline, μg/mL	14.07(4.84,24.53)	15.11(5.82,26.19)	0.239
Current smoking, n (%)	60(30.15)	79(39.70)	0.043
Current drinking, n (%)	55(27.64)	57(28.64)	0.967
Treatment group, n (%)			
Enalapril	102 (51.26)	102 (51.26)	1.000
Enalapril-folic acid	97 (48.74)	97 (48.74)	
MTHFR C677T, n (%)			
CC	56 (28.14)	56 (28.14)	0.521
CT	102 (51.26)	93 (46.73)	
TT	41 (20.60)	50 (25.13)	

BMI, body mass index; SBP, systolic blood pressure; DBP, diastolic blood pressure; TG, triglycerides; TC, total cholesterol; SUA, serum uric acid; HDL-C, high-density lipoprotein cholesterol; FBG, fasting blood glucose; HCY, homocysteine; and MTHFR, methylenetetrahydrofolate reductase.

### Association of plasma choline with the risk of cancer

**Figure 2:**
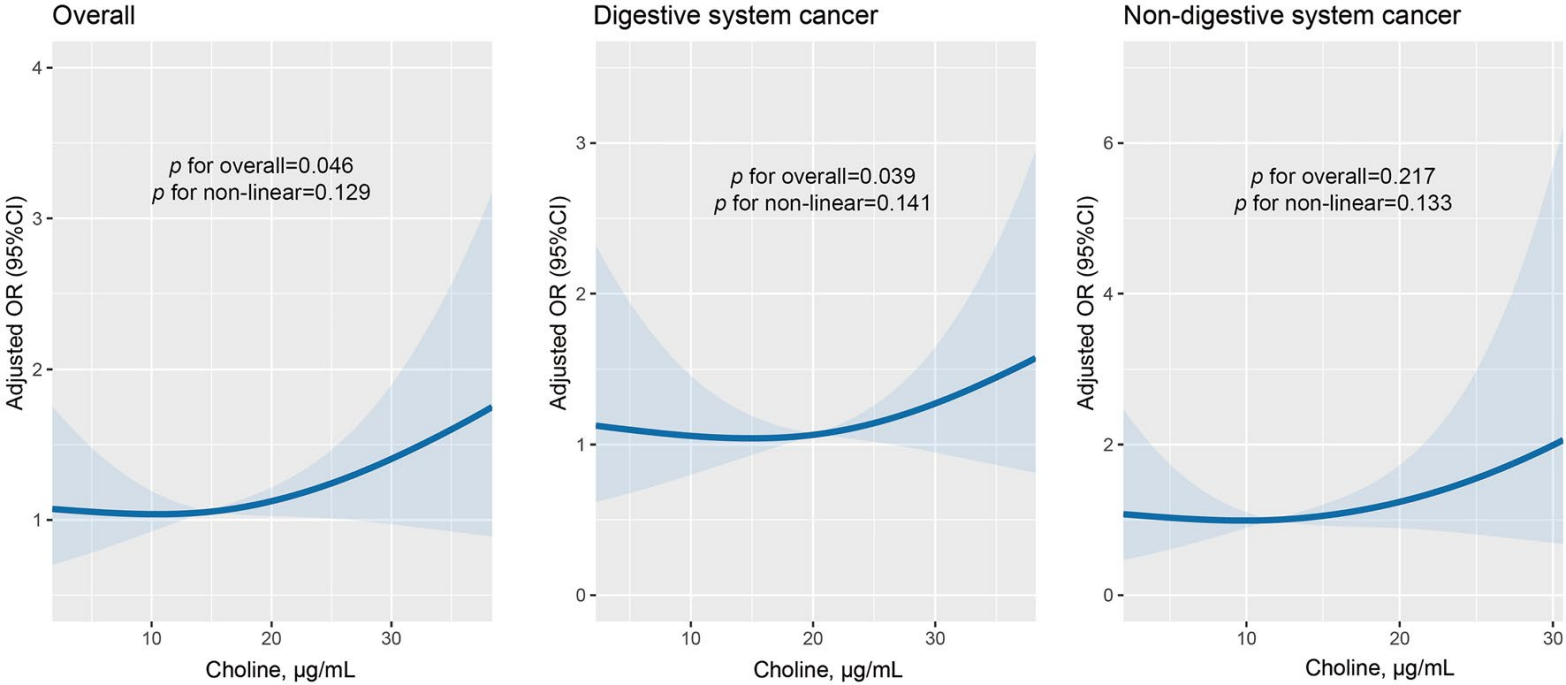
Association between serum choline levels (per SD) and cancer risk using RCS. *Note* Models were adjusted for BMI, treatment group, smoking status, alcohol drinking, MTHFR C677T, systolic blood pressure, folate, triglycerides, cholesterol, uric acid, glucose, and homocysteine.

The median follow-up time was 3.91 years (interquartile range, 2.08, 4.29 years). Figure 2 illustrates the association of serum choline concentrations with overall, digestive system, and non-digestive system cancer risk. A positive dose–response association between serum choline levels and the risk of overall and digestive system cancer in the study population was observed. Table 2 presents the crude and adjusted ORs (95%CI) for the association between serum choline and overall cancer risk. Each SD increment of serum choline concentration significantly elevated the overall cancer risk (OR = 1.51, 95%CI 1.02–2.24). Compared with participants in the lowest choline quartile (Q1 group), those in the highest quartile (Q4) had a 3.69-fold increased risk of cancer in the adjusted models (OR = 3.69, 95% CI 1.17–11.63). We further explored the effect of serum choline levels on the occurrence of digestive system and non-digestive system cancer (Table 3). The positive association between serum choline levels and digestive cancer risk was observed, with corresponding ORs (95%CI) of 1.79 (1.07–2.97, [per SD increase]) and 6.01(1.14–31.67, [Q4 vs. Q1]), respectively.

**Table 2:** The association of choline with the risk of incident total cancer.

Choline (μg/mL)	Cases/controls	Crude model		Adjusted model	
		OR (95%CI)	*p*-value	OR (95%CI)	*p*-value
Per SD (11.49 μg/mL)	199/199	1.46(1.00,2.12)	0.051	**1.51(1.02,2.24)**	**0.040**
Quartiles					
Q1 (< 5.35)	46/55	Ref.		Ref.	
Q2 (5.35–14.70)	50/48	1.29(0.75,2.24)	0.359	1.27(0.69,2.32)	0.444
Q3 (14.71–25.18)	48/51	1.96(0.82,4.68)	0.130	2.31(0.88,6.09)	0.091
Q4 (≥ 25.19)	55/45	**3.11(1.09,8.86)**	**0.034**	**3.69(1.17,11.63)**	**0.026**

Models were adjusted for BMI, treatment group, smoking status, alcohol drinking, MTHFR C677T, systolic blood pressure, folate, triglycerides, cholesterol, uric acid, glucose, and homocysteine.

Statistically significant values are shown in bold with *p* value < 0.05.

**Table 3:** The association of choline with the digestive system and non-digestive system cancer risk.

Choline (μg/mL)	Digestive system cancer			Non-digestive system cancer		
	Cases/controls	OR (95%CI)	*p*-value	Cases/controls	OR (95%CI)	*p*-value
Per SD (11.49 μg/mL)	113/113	**1.79(1.07,2.97)**	**0.025**	86/86	1.46(0.67,3.19)	0.345
Quartiles						
Q1	23/34	Ref.		21/22	Ref.	
Q2	32/24	2.32(0.82,6.59)	0.114	22/21	1.47(0.49,4.47)	0.494
Q3	25/31	3.38(0.66,17.34)	0.145	22/22	1.27(0.40,4.02)	0.682
Q4	33/24	**6.01(1.14,31.67)**	**0.034**	21/21	2.02(0.38,10.77)	0.411

Models were adjusted for BMI, treatment group, smoking status, alcohol drinking, MTHFR C677T, systolic blood pressure, folate, triglycerides, cholesterol, uric acid, glucose, and homocysteine.

The cutoffs of serum choline in the digestive were 6.42, 20.24, and 28.09.

The cutoffs of serum choline in the non-digestive system were 4.25, 11.76, and 19.24.

Statistically significant values are shown in bold with *p* value < 0.05.

**Figure 3:**
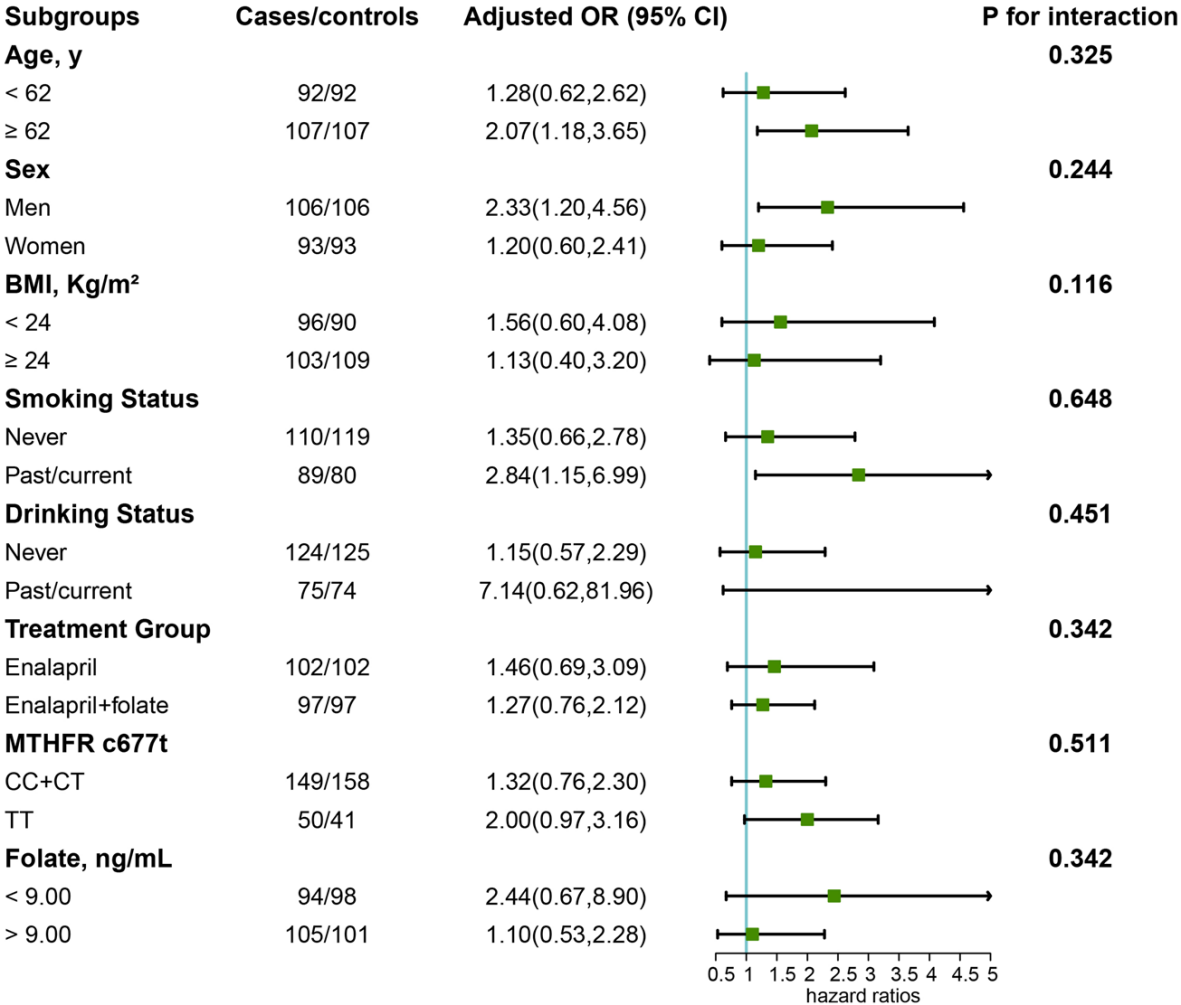
Stratified analysis of the association of serum choline levels (per SD) with the risk of cancer. *Note* Models were adjusted for BMI, treatment group, smoking status, alcohol drinking, MTHFR C677T, systolic blood pressure, folate, triglycerides, cholesterol, uric acid, glucose, and homocysteine, except for the variables used for stratification in each subgroup analysis.

Figure 3 shows the effects of serum choline on overall cancer risk across various subgroups defined by potential risk factors. Notable subgroup differences were observed. For instance, serum choline (per SD, 11.49 μg/mL) was associated with a higher risk of overall cancer among participants who were older (compared to relatively younger participants with a median age of 62 years), male (compared to female), and smokers (compared to non-smokers). However, none of the tests for interactions between choline and any of the stratified subgroups were statistically significant.

In the sensitivity analysis, after excluding 25 cancer cases that occurred within the first year of follow-up, the association remained statistically significant after further adjustments for potential confounders (Table 4).

**Table 4:** Sensitivity analysis on the association of choline and overall cancer risk after excluding 25 cases occurring within one year of follow-up.

Choline (μg/mL)	Cases/controls	Crude model			Adjusted model	
		OR (95%CI)	*p*-value		OR (95%CI)	*p*-value
Per SD (11.49 μg/mL)	174/199	1.55(1.04,2.32)	0.033		1.65(1.08,2.53)	0.020
Quartiles of choline						
Q1	44/55	Ref			Ref	
Q2	39/48	1.09(0.61,1.96)	0.008		1.02(0.53,1.96)	0.945
Q3	41/51	2.02(0.80,5.12)	0.669		2.36(0.86,6.49)	0.097
Q4	50/45	**3.65(1.20,11.06)**	**0.022**		**4.32(1.30,14.37)**	**0.017**

Models were adjusted for BMI, treatment group, smoking status, alcohol drinking, MTHFR C677T, systolic blood pressure, folate, triglycerides, cholesterol, uric acid, glucose, and homocysteine.

Statistically significant values are shown in bold with all *p* values < 0.05.

## Discussion

The primary observation from this prospective, case–control study nested within the CSPPT is the elevated risk of incident cancer in hypertensive patients with higher serum choline levels. In subgroup analyses, similar associations were observed among older patients, males, and smokers. Sensitivity analyses, which excluded cancer cases that occurred within the first year, further confirmed the robustness of our findings.

Despite the limited data on serum choline concentrations in relation to cancer risk from previous studies, our findings align with those of several other prospective studies. A case–control study nested within the Women's Health Initiative Observational Study, a prospective cohort study designed to investigate the predictors and causes of morbidity and mortality, found that plasma choline was positively associated with the risk of rectal cancer in postmenopausal women^20^. A nested case–control study of male smokers in the Alpha-Tocopherol, Beta-Carotene Cancer Prevention (ATBC) Study observed that men with higher serum choline had approximately a threefold greater risk of developing colorectal cancer over a 14-year period in the fully adjusted models^19^. However, a recent case–control study nested within the EPIC cohort observed an inverse association between plasma choline and colorectal cancer risk, which contrasts with our findings^22^. Another nested case–control study with 297 incident cases and 631 matched controls from a cohort of 18,244 men in Shanghai reported an inverse association between choline and vitamin B6 and the risk of hepatocellular carcinoma development^23^. A case–control study (129 cases vs. 258 controls) nested within the Shanghai Cohort study found that elevated serum choline decreased the risk of pancreatic cancer^24^. While some studies have reported contradictory results, others have found no significant associations between serum choline and malignant tumors. A prospective case–control study involving 613 cases and 1,190 matched controls nested within the population-based Northern Sweden Health and Disease Study found no clear association between methionine and colorectal cancer risk^25^. Similarly, de Vogel et al.^26^ did not find a significant association between serum choline and distal colorectal adenomas in a cross-sectional study involving 10,601 healthy subjects.

It’s worth noting that the discrepancies in findings across these studies could be attributed to several factors. Regional differences, for instance, might play a role, as dietary habits, environmental exposures, and genetic predispositions can vary significantly across different geographical areas. Additionally, the specific populations studied may have inherent differences that influence the relationship between serum choline and cancer risk. For example, variations in age, gender, health status, and other demographic factors could potentially modulate the effects of choline on cancer development. Furthermore, differences in study design, such as the duration of follow-up, the methods used to measure choline levels, and the criteria for case selection, could also contribute to the observed inconsistencies. It's essential to consider these factors when interpreting and comparing the results of various studies on this topic. In our study, we focused on the serum choline concentration in a hypertensive population, with a median value of 14.71 μg/mL. When comparing this data with findings from other studies, it’s evident that the serum choline concentrations fluctuate between 10 and 23 μg/mL in other studies^20,24–26^. Thus, the serum choline level in our hypertensive cohort is not significantly different from that of non-hypertensive populations. We believe this comparison provides a comprehensive perspective on the serum choline levels across different populations. Future research should use a larger sample size and a longer follow-up period to substantiate this difference.

Several notable differences emerged from our study’s subgroup analyses (Fig. 3), and we attempt to explain them as follows. First, continuous exposure to risk factors, including elevated serum choline, is likely to increase the risk of incident cancer, which may explain why a significant association was observed predominantly among older participants^27^. Second, although abnormally elevated estrogen concentrations are associated with an increased risk of breast and gynecologic cancers (cervical, endometrial, and ovarian), estrogen has a protective effect against certain digestive cancers (liver, colorectal, and esophageal) and lung cancer. This observation from several epidemiological studies might help explain why a positive association was predominantly observed in males^28–30^. Third, cigarettes and other tobacco products are estimated to account for 2.4 million cancer-related deaths worldwide annually^2^, Further studies should explore the combined influence of smoking and choline on the development of malignant tumors.

Controversies about the genuine effect of serum choline on the cancer risk remain. However, epidemiological evidence for the role of dietary choline in cancer development and progression has increased in the last decade, mainly due to the creation of a food composition database for choline in 2003. Zhang et al.^16^ suggested that consumption of choline was inversely associated with the risk of breast cancer in a case–control study with 807 cases and 807 age- and residence- matched controls. Another case–control study demonstrated an inverse association between total choline intake with colorectal cancer risk in both men and women, and the inverse associations were not modified by folate intake^17^. Similarly, a beneficial effect of choline intake on the carcinogenesis of nasopharyngeal carcinoma was observed in another case–control study^18^. A meta-analysis, which included 16 studies on choline intake, concluded that high intake of dietary choline was associated with a lower risk of cancer incidence, especially for colorectal cancer^31^.

The relationship between dietary choline intake and serum choline concentrations has long been a controversial question. Previous studies have found that levels of supplemental and dietary choline did not correlate with serum choline levels in healthy, elderly participants^32^. However, Hirsch et al.^33^ have found that consumption of a diet rich in choline caused prolonged elevations in serum choline concentrations, in a study including sixteen healthy subjects. Differences in study design and study populations might explain these discrepancies.

The potential mechanism surrounding the association of elevated serum choline with increased risk of cancer remains uncertain. A prior study has proven that choline kinase, an enzyme that initiates the first step and the rate-limiting step for converting choline to phosphatidylcholine, is overexpressed in human colorectal cancer cells^34^. Choline kinase is a prospective new focus for cancer treatment. Malignancy and heightened cellular proliferation are associated with choline kinase-α expression/activity^35^. Colonic phenotype, which is an increase of total choline-containing compounds, has recently been recognized as a metabolic sign of malignancy^36^. Positron emission tomography can measure differential uptake of choline^37^, and has been observed in some cancers. Yet other studies have suggested that increased choline metabolism is due to the malignancy itself, as opposed to being a byproduct of enhanced proliferation^38^. In our study, the elevated risk of cancer with higher serum choline levels persisted even after excluding cases that had occurred within the first year of follow-up; while still plausible, it is unlikely that any increase in growth of precancerous lesions due to choline is reflected by our risk estimates.

The major strength of the study lies in its prospective design, which allows for a temporal relationship between exposure and outcome to be established, reducing the likelihood of recall bias, and providing a more robust framework for causality inference compared to retrospective studies. Another strength is the broad assessment of potential confounders which were rarely evaluate in previous studies. However, limitations should also be noted. First, serum choline concentrations were only assessed once at baseline to evaluate long-term exposure risk. An average of repeated baseline readings would provide more accuracy. Second, we did not have any detailed dietary information about choline intake. Third, the participants in the present study were exclusively hypertensive patients. This introduces potential selection bias and raises concerns about the generalizability of our findings, especially when considering populations without hypertension or non-Chinese groups. While we adjusted for blood pressure in the multivariate analysis, the potential bias due to hypertension cannot be entirely disregarded. Additionally, the effect of unmeasured confounding factors further complicates the interpretation of our results. Fourth, the relationship of serum choline levels with precise cancer sites was difficult to assess, as the relatively small sample size only provided a small number of site-specific cancers. Finally, in this study, we did not assess the effects of dietary intake (specifically total energy intake), family history of cancer, personal history of diabetes, and physical activity on the association between serum choline and cancer risk. The absence of these data may influence the interpretation of our findings, and this limitation should be considered when drawing conclusions from the results.

## Conclusion

We have found a positive association between elevated levels of serum choline with increased risk of incident cancer. Although preliminary, our findings have critical clinical implications for cancer prevention and diagnosis.

## References

[1] Bray F, Ferlay J, Soerjomataram I, Siegel RL, Torre LA, Jemal A (2018). Global cancer statistics 2018: GLOBOCAN estimates of incidence and mortality worldwide for 36 cancers in 185 countries. CA Cancer J. Clin..

[2] GBD 2017 Risk Factor Collaborators. Global, regional, and national comparative risk assessment of 84 behavioural, environmental and occupational, and metabolic risks or clusters of risks for 195 countries and territories, 1990–2017: A systematic analysis for the Global Burden of Disease Study 2017. *Lancet* **392**(10159), 1923–1994 (2018). 10.1016/S0140-6736(18)32225-6 PMC6227755 30496105

[3] de Martel C, Georges D, Bray F, Ferlay J, Clifford GM (2020). Global burden of cancer attributable to infections in 2018: A worldwide incidence analysis. Lancet Glob. Health.

[4] Pflaum T, Hausler T, Baumung C, Ackermann S, Kuballa T, Rehm J (2016). Carcinogenic compounds in alcoholic beverages: An update. Arch. Toxicol..

[5] Wiseman M (2008). The second World Cancer Research Fund/American Institute for Cancer Research expert report. Food, nutrition, physical activity, and the prevention of cancer: A global perspective. Proc. Nutr. Soc..

[6] Singh RK, Chang HW, Yan D, Lee KM, Ucmak D, Wong K (2017). Influence of diet on the gut microbiome and implications for human health. J. Transl. Med..

[7] World Health Organization. Evaluation of certain contaminants in food. *World Health Organ Tech. Rep. Ser.* (1002), 1–166 (2017). 29144071

[8] Zeisel SH, Klatt KC, Caudill MA (2018). Choline. Adv. Nutr..

[9] Zeisel SH (2006). Choline: Critical role during fetal development and dietary requirements in adults. Annu. Rev. Nutr..

[10] Zeisel SH (2009). Importance of methyl donors during reproduction. Am. J. Clin. Nutr..

[11] Fischer LM, daCosta KA, Kwock L, Stewart PW, Lu TS, Stabler SP (2007). Sex and menopausal status influence human dietary requirements for the nutrient choline. Am. J. Clin. Nutr..

[12] Institute of Medicine Standing Committee on the Scientific Evaluation of Dietary Reference, I., O.B.V. its Panel on Folate, and Choline, The National Academies Collection: Reports funded by National Institutes of Health. In *Dietary Reference Intakes for Thiamin, Riboflavin, Niacin, Vitamin B(6), Folate, Vitamin B(12), Pantothenic Acid, Biotin, and Choline*. 1998, National Academies Press (US) Copyright © 1998, National Academy of Sciences: Washington (DC).

[13] Kohlmeier M, da Costa KA, Fischer LM, Zeisel SH (2005). Genetic variation of folate-mediated one-carbon transfer pathway predicts susceptibility to choline deficiency in humans. Proc. Natl. Acad. Sci. U. S. A..

[14] Niculescu MD, Zeisel SH (2002). Diet, methyl donors and DNA methylation: Interactions between dietary folate, methionine and choline. J. Nutr..

[15] Kim YI (1999). Folate and carcinogenesis: Evidence, mechanisms, and implications. J. Nutr. Biochem..

[16] Zhang CX, Pan MX, Li B, Wang L, Mo XF, Chen YM (2013). Choline and betaine intake is inversely associated with breast cancer risk: A two-stage case-control study in China. Cancer Sci..

[17] Lu MS, Fang YJ, Pan ZZ, Zhong X, Zheng MC, Chen YM (2015). Choline and betaine intake and colorectal cancer risk in Chinese population: A case-control study. PLoS ONE.

[18] Zhou RF, Chen XL, Zhou ZG, Zhang YJ, Lan QY, Liao GC (2017). Higher dietary intakes of choline and betaine are associated with a lower risk of primary liver cancer: A case-control study. Sci. Rep..

[19] Guertin KA, Li XS, Graubard BI, Albanes D, Weinstein SJ, Goedert JJ (2017). Serum trimethylamine N-oxide, carnitine, choline, and betaine in relation to colorectal cancer risk in the alpha tocopherol, beta carotene cancer prevention study. Cancer Epidemiol. Biomark. Prev..

[20] Bae S, Ulrich CM, Neuhouser ML, Malysheva O, Bailey LB, Xiao L (2014). Plasma choline metabolites and colorectal cancer risk in the Women's Health Initiative Observational Study. Cancer Res..

[21] Huo Y, Li J, Qin X, Huang Y, Wang X, Gottesman RF (2015). Efficacy of folic acid therapy in primary prevention of stroke among adults with hypertension in China: The CSPPT randomized clinical trial. JAMA.

[22] Nitter M, Norgård B, de Vogel S, Eussen SJ, Meyer K, Ulvik A (2014). Plasma methionine, choline, betaine, and dimethylglycine in relation to colorectal cancer risk in the European Prospective Investigation into Cancer and Nutrition (EPIC). Ann. Oncol..

[23] Butler LM, Arning E, Wang R, Bottiglieri T, Govindarajan S, Gao YT (2013). Prediagnostic levels of serum one-carbon metabolites and risk of hepatocellular carcinoma. Cancer Epidemiol. Biomark. Prev..

[24] Huang JY, Luu HN, Butler LM, Midttun Ø, Ulvik A, Wang R (2020). A prospective evaluation of serum methionine-related metabolites in relation to pancreatic cancer risk in two prospective cohort studies. Int. J. Cancer.

[25] Myte R, Gylling B, Schneede J, Ueland PM, Häggström J, Hultdin J (2016). Components of one-carbon metabolism other than folate and colorectal cancer risk. Epidemiology.

[26] de Vogel S, Schneede J, Ueland PM, Vollset SE, Meyer K, Fredriksen A (2011). Biomarkers related to one-carbon metabolism as potential risk factors for distal colorectal adenomas. Cancer Epidemiol. Biomark. Prev..

[27] Beckett NS, Peters R, Fletcher AE, Staessen JA, Liu L, Dumitrascu D (2008). Treatment of hypertension in patients 80 years of age or older. N. Engl. J. Med..

[28] Gallus S, Bosetti C, Franceschi S, Levi F, Simonato L, Negri E (2001). Oesophageal cancer in women: Tobacco, alcohol, nutritional and hormonal factors. Br. J. Cancer.

[29] Schabath MB, Wu X, Vassilopoulou-Sellin R, Vaporciyan AA, Spitz MR (2004). Hormone replacement therapy and lung cancer risk: A case-control analysis. Clin. Cancer Res..

[30] Slattery ML, Ballard-Barbash R, Edwards S, Caan BJ, Potter JD (2003). Body mass index and colon cancer: An evaluation of the modifying effects of estrogen (United States). Cancer Causes Control.

[31] Youn J, Cho E, Lee JE (2019). Association of choline and betaine levels with cancer incidence and survival: A meta-analysis. Clin. Nutr..

[32] Sanchez CJ, Hooper E, Garry PJ, Goodwin JM, Goodwin JS (1984). The relationship between dietary intake of choline, choline serum levels, and cognitive function in healthy elderly persons. J. Am. Geriatr. Soc..

[33] Hirsch MJ, Growdon JH, Wurtman RJ (1978). Relations between dietary choline or lecithin intake, serum choline levels, and various metabolic indices. Metabolism.

[34] Ramírez de Molina A, Rodríguez-González A, Gutiérrez R, Martínez-Piñeiro L, Sánchez J, Bonilla F (2002). Overexpression of choline kinase is a frequent feature in human tumor-derived cell lines and in lung, prostate, and colorectal human cancers. Biochem. Biophys. Res. Commun..

[35] Bagnoli M, Granata A, Nicoletti R, Krishnamachary B, Bhujwalla ZM, Canese R (2016). Choline metabolism alteration: A focus on ovarian cancer. Front. Oncol..

[36] Glunde K, Bhujwalla ZM, Ronen SM (2011). Choline metabolism in malignant transformation. Nat. Rev. Cancer.

[37] Vander Heiden MG (2011). Targeting cancer metabolism: A therapeutic window opens. Nat. Rev. Drug Discov..

[38] Aboagye EO, Bhujwalla ZM (1999). Malignant transformation alters membrane choline phospholipid metabolism of human mammary epithelial cells. Cancer Res..

